# Functional characterization of the chlorzoxazone 6-hydroxylation activity of human cytochrome P450 2E1 allelic variants in Han Chinese

**DOI:** 10.7717/peerj.9628

**Published:** 2020-07-31

**Authors:** Ting Wang, Huihui Du, Jingsong Ma, Lu Shen, Muyun Wei, Xianglong Zhao, Luan Chen, Mo Li, Guorong Li, Qinghe Xing, Lin He, Shengying Qin

**Affiliations:** 1 Bio-X Institutes, Shanghai Jiaotong University, Shanghai, China; 2 School of Life Sciences, Shandong Normal University, Shandong, China; 3 Institutes of Biomedical Sciences, Fudan University, Shanghai, China; 4 Baoan Maternal and Child Health Hospital, Jinan University, Guangdong, China; 5 Collaborative Innovation Center, Jining Medical University, Shandong, China

**Keywords:** Cytochrome P450 2E1 (CYP2E1), Genetic polymorphisms, Chlorzoxazone, Metabolism, Enzyme activity

## Abstract

**Backgrounds:**

Cytochrome P450 (P450) 2E1 is one of the primary enzymes responsible for the metabolism of xenobiotics, such as drugs and environmental carcinogens. The genetic polymorphisms of the *CYP2E1* gene in promoter and coding regions have been identified previously in the Han Chinese population from four different geographic areas of Mainland China.

**Methods:**

To investigate whether genetic variants identified in the *CYP2E1* coding region affect enzyme function, the enzymes of four single nucleotide polymorphism (SNP) variants in the coding region (novel c.1009C>T, causing p.Arg337X, where X represents the translational stop codon; c.227G>A, causing p.Arg76His; c.517G>A, yielding p.Gly173Ser; and c.1263C>T, presenting the highest allele frequency), two novel alleles (c.[227G>A;1263C>T] and c.[517G>A;1263C>T]), and the wild-type *CYP2E1* were heterologously expressed in COS-7 cells and functionally characterized in terms of expression level and chlorzoxazone 6-hydroxylation activity. The impact of the CYP2E1 variant sequence on enzyme activity was predicted with three programs: Polyphen 2, PROVEAN and SIFT.

**Results:**

The prematurely terminated p.Arg337X variant enzyme was undetectable by western blotting and inactive toward chlorzoxazone 6-hydroxylation. The c.1263C>T and c.[517G>A;1263C>T] variant enzymes exhibited properties similar to those of the wild-type CYP2E1. The *CYP2E1* variants c.227G>A and c.[227G>A;1263C>T] displayed significantly reduced enzyme activity relative to that of the wild-type enzyme (decreased by 42.8% and 32.8%, respectively; *P* < 0.01). The chlorzoxazone 6-hydroxylation activity of the c.517G>A transfectant was increased by 31% compared with the wild-type CYP2E1 enzyme (*P* < 0.01). Positive correlations were observed between the protein content and enzyme activity for CYP2E1 (*P* = 0.0005, *r*^2^ = 0.8833). The characterization of enzyme function allelic variants in vitro was consistent with the potentially deleterious effect of the amino acid changes as determined by prediction tools.

**Conclusions:**

These findings indicate that the genetic polymorphisms of *CYP2E1,* i.e., c.1009C>T (p.Arg337X), c.227G>A (p.Arg76His), and c.517G>A (p.Gly173Ser), could influence the metabolism of CYP2E1 substrates, such as chlorzoxazone.

## Introduction

The cytochrome P450 (CYP) supergene family consists of 57 functional genes and 58 pseudogenes (Uehara et al., 2015) and encodes enzymes that catalyze the conversion of heterogeneous biochemical substances (e.g., drugs and toxic chemicals) and endogenous biochemical substances (e.g., industrial solvents and pollutants) into active metabolites. In addition to detoxification, these enzymes often catalyze the metabolic activity of carcinogens, thus allowing these chemicals to reach their ultimate carcinogenic form (Gonzalez, 1990; Shimada et al., 1994; Nelson et al., 1996; Rendic & Di Carlo, 1997). While genetic variants contribute to function variations for genes encoding drug metabolizing enzymes, drug transporters, drug receptors, and signaling molecule genes that encode drug metabolizing enzymes have received the most attention for clinical implementation (Weinshilboum & Wang, 2017). Many studies have shown that the non-synonymous single-nucleotide polymorphisms (SNPs) of genes encoding drug-metabolizing enzymes are significantly correlated with the efficacy of clinical drugs (Sim, Kacevska & Ingelman-Sundberg, 2013). By influencing variations in the pharmacokinetics and pharmacodynamics, the genetic heterogeneity among these genes contributes to population heterogeneity in drug response (Hasler, 1999; Dresser, Spence & Bailey, 2000; Kim et al., 2008).

The human cytochrome P450 family 2 subfamily E polypeptide 1 (CYP2E1) gene is located on chromosome 10q24.3-qter and contains nine exons and eight introns (Umeno et al., 1988). The *CYP2E1* gene encodes a phase I metabolic enzyme that is mainly expressed in liver tissues and exhibits a number of important functions (Lieber, 1997). One notable function is its ability to accelerate ethanol-induced hepatotoxicity. Another critical function is its ability to metabolize a variety of small-molecule compounds, such as chlorzoxazone and isoniazid, as well as potential carcinogens, including benzene and styrene (Ingelman-Sundberg et al., 1994; Lu & Cederbaum, 2008).

Many *CYP2E1* alleles have been identified thus far, with the protein products demonstrating decreased, deficient or normal enzyme activity (Hanioka et al., 2010). The frequency distribution of different *CYP2E1* alleles in different poulations has been found to vary widely across different ethnic cohorts (Ulusoy, Arinc & Adali, 2007). Due to the widespread existence of such population differences, it has been suspected that population-specific sequencing-based genetic heterogeneity may account for differences in the drug responses of interest (Kongruttanachok et al., 2001; Lee et al., 2001; Haufroid et al., 2002; Xu et al., 2016). Recent investigations have demonstrated that the distributions of nucleotide polymorphisms or haplotypes in specific genes tend to be regional. For instance, CYP2E1*1C has been found to exhibit a relatively high frequency in Caucasians (∼98.9%) but lower frequency in the Thai population (Kongruttanachok et al., 2001; Howard et al., 2003). In addition, CYP2E1*1D has been commonly found in Indo-Asians at a higher frequency (∼31.2%) than in the Swedish population (1.1%) (Hu et al., 1999; Howard et al., 2003). Alterations of CYP2E1 enzyme activity represent an important contributor to the interindividual differences in drug response. Because CYP2E1 phenotypic polymorphisms partly account for interindividual differences in drug metabolism and severe adverse drug reactions (ADRs), functional characterization of CYP2E1 allelic variants is of great importance in implementing individualized drug treatment.

Studies have shown that the existence of *CYP2E1* polymorphisms is significantly related to CYP2E1 activity, disease susceptibility and xenobiotic toxicity (Hu et al., 1999; Novak & Woodcroft, 2000). Watanabe, Hayashi & Kawajiri (1994) reported on the regulation and expression of CYP2E1*1C and CYP2E1*2C in the *CYP2E1* 5′-flanking region and verified that the expression level of *CYP2E1* mRNA in subjects carrying genotype B (c1/c2) who drink alcohol daily was approximately 2.0 times higher than that in nondrinkers with genotype A (c1/c1) (Watanabe, Hayashi & Kawajiri, 1994). Previous studies have shown that there are great differences in the protein and enzyme activity of CYP2E1 between individuals, which is similar to the trends in other P450 enzyme members (Neafsey et al., 2009). In the *CYP2E1* coding region, four single nucleotide polymorphism (SNP) variants: c.227G>A, p.Arg76His, which is CYP2E1*2; c.517G>A, which yields a p.Gly173Ser exchange; c.1009C>T which carries a novel mutation that causes the p.Arg337X (X represents the translational stop codon) with amino acid substitution; c.1263C>T, which has the highest allele frequency, and two novel alleles (c.[227G>A;1263C>T], which yields a p. Arg76His exchange and c.[517G>A;1263C>T], which causes p.Gly173Ser) have been identified in the Han Chinese population (Tang et al., 2010). Many functional analyses of *CYP2E1* alleles have been performed. For instance, Hu et al. (1999) investigated the effect of *CYP2E1* alleles causing amino acid substitutions (CYP2E1*2, CYP2E1*3 and wild-type CYP2E1*1) on chlorzoxazone hydroxylation using the recombinant CYP2E1 enzymes of the wild type (CYP2E1.1) and variants (CYP2E1.2 with Arg76His and CYP2E1.3 with Val389Ile) expressed in COS-1 cells and suggested that the protein content and catalytic activity of CYP2E1.2 were only 37% and 36% of those of CYP2E1.1, respectively, while the CYP2E1*3 cDNA variant was indistinguishable from the wild-type cDNA for all variables investigated (Hu et al., 1997). Subsequently, Hanioka et al. (2003) demonstrated that the protein level of CYP2E1.2 was reduced to 29% compared with that of CYP2E1.1 (Hanioka et al., 2003). Zhu et al. (2018) used variant effect prediction tools to determine the potentially deleterious effect of the amino acid changes on protein function and demonstrated that the Gly173Ser variant may affected by the corresponding amino acid substitutions (Zhu et al., 2018). However, there are still few reports on the effects of the genetic polymorphisms of *CYP2E1* on the detailed enzymatic properties or about comparisons among the identified allelic variants with amino acid substitutions. Moreover, there is a lack of phenotype profile of *CYP2E1* allelic variants, especially variants identified in the same ethnic population, to support the optimal pharmacotherapy of drug treatment, particularly for the appropriate dosing of drugs with a narrow therapeutic index, such as chlorzoxazone.

Chlorzoxazone, a muscle relaxant drug, has been widely used as a probe substrate to measure CYP2E1 activity via hydroxylation of chlorzoxazone *in vivo* and *in vitro* (Lucas et al., 1995; Lucas, Menez & Berthou, 1996). Chlorzoxazone 6-hydroxylation has been suggested to be a typical reaction catalyzed by CYP2E1 in human liver microsomes (Peter et al., 1990; Kim & O’shea, 1995). Therefore, we functionally characterized CYP2E1 allelic variants *in vitro* using chlorzoxazone as a representative substrate.

The present study was designed to determine the functional effect of *CYP2E1* allelic variants found in the Han Chinese population. To achieve this, we utilized a standard approach to prepare recombinant CYP2E1 enzymes by transfecting COS-7 cells with the cDNA of six *CYP2E1* allelic variants (c.227G>A rs72559710; c.517G>A rs60452492; novel c.1009C>T; c.1263C>T rs2515641; c.[227G>A;1263C>T]; and c.[517G>A;1263>T]) and wild-type *CYP2E1*, and studied the effect of those enzymes on protein level and enzyme activity toward chlorzoxazone 6-hydroxylation using chlorzoxazone as a probe substrate. Computational methods have also been developed in an attempt to predict the effect of genetic variation in encoded amino acid sequence on protein stability and enzyme activity. Studies of these variants showed functional effects that might indicate clinical utility with regard to variations in drug response phenotypes. The information gained in this study should help to promote a better understanding of CYP2E1-mediated drug metabolism.

## Materials & Methods

### Construction of CYP2E1 expression plasmids

The native CYP2E1 cDNA in pUC19 plasmid was obtained from Qinglan Technologies (Shanghai, China), released by digestion with *Eco* R I and *Kpn* I (Thermo Scientific, Beijing, China), and then subcloned into pcDNA3.1 (±) vector (Invitrogen, Carlsbad, CA, USA). Site-directed mutagenesis was then performed using the QuikChange site-directed mutagenesis kit (Agilent Technologies, Santa Clara, CA) according to the manufacturer’s instructions using wild-type CYP2E1 plasmid as a template to create expression constructs for each of the variants to be studied (Table 1). Sequences of the primers used to perform site-directed mutagenesis are listed in the Table S1, and all CYP2E1 variant plasmids were sequenced to confirm successful mutagenesis. The DNA concentration and quality were evaluated with Nano Drop 2000 UV-Vis Spectrophotometer (Thermo, Wilmington, DE, USA).

**Table 1:** Selected variants of *CYP2E1* exon regions in the Han Chinese population. (A) Wild-type. (B) The accession number for the protein reference sequence is P05181. (C) The frequencies of the alleles, data from Bio-X database of *CYP2E1* gene polymorphisms for normal Chinese Han subjects.

Variant name	Nucleotide change	Effect^B^	Frequency (%)^C^
WT^A^	None	None	49.55
c.227G>A	227G>A	76Arg-His	0.2
c.517G>A	517G>A	173Gly-Ser	0.1
c.1263C>T	1263C>T	None	15.1
c.1009C>T	1009C>T	337Arg-Stop	0.5
c.[227G>A+1263C>T]	1263C>T; 227G>A	76Arg-His+None	3.2
c.[517G>A+1263C>T]	1263C>T; 517G>A	173Gly-Ser+None	1.5

### Timely transfection and expression of CYP2E1-encoding plasmids in COS-7 cells

We selected COS-7 African simian kidney cancer cells for our expression studies because they do not express the CYP2E1. The cells were seeded on 10 cm culture dishes (ATCC, VA, USA) in Dulbecco’s modified Eagle’s medium (DMEM) containing 10% Fetal bovine serum (FBS; PAA, Piscataway, NJ, USA) and cultured at 37 °C under 5% CO_2_ overnight. Subsequently, a plasmid transfection experiment was performed when cells were ∼90% confluent and plasmids carrying desired CYP2E1 cDNAs were transfected into COS-7 cells under optimal transfection condition with Lipofectamine 2000 (Invitrogen, Carlsbad, CA) according to the manufacturer’s instructions. After 6 h of incubation at 37 °C, the culture medium was replaced with DMEM containing 10% FBS and the cells were incubated for an additional 48 h. The cells were then scrapped from the culture dishes, washed twice with PBS (pH 7.4), and then resuspended in PBS (pH 7.4) containing 0.2 mM EDTA, 1 mM dithiothreitol, and 20% glycerol. After sonication for 20 s, the homogenate was centrifuged at 9000 g and 4 °C for 20 min. The resulting supernatant was transferred to a new tube and centrifuged at 105,000 g and 4 °C for 60 min. Subsequently, the microsomal fraction was resuspended in 0.25 M sucrose and the protein content was measured using the Pierce BCA Protein Assay Kit (Thermo Fisher Scientific Catalog No. 23225) according to the manufacturer’s guidelines.

### Western blot analysis

Total CYP2E1 protein levels in COS-7 cells microsomal fractions were determined by western blot analysis. The proteins were separated by 10% sodium dodecyl sulfate-polyacrylamide gel electrophoresis and electroblotted onto a polyvinylidene difluoride membrane (Thermo, CA, USA). After incubation with the rabbit anti-human CYP2E1 primary antibody (Sigma-Aldrich [MO, USA], Cat. No. HPA009128) for 24 h at 4°C, the PVDF membranes were incubated with the HRP-conjugated anti-rabbit IgG (Sigma-Aldrich) for 2 h at room temperature. The expression of each variant was normalized with regard to that of the wild-type CYP2E1. Proteins were detected using the SuperSignal™ West Dura Extended Duration Substrate (Thermo Scientific Catalog No. 34075), and images were captured on a Tanon-5200Multi Chemiluminescent Imaging System (Tanon, Shanghai, China). Quantification of protein density was performed with Image J software (https://imagej.nih.gov/ij/download.html).

### Enzyme activity assays

CYP2E1 enzyme kinetic parameters were characterized with chlorzoxazone as a substrate. Chlorzoxazone 6-hydroxylation activities were determined by high-performance liquid chromatography (HPLC) as described previously with some modifications (Hanioka et al., 2003). Specifically, the microsomal fractions of COS-7 cells were incubated in a 2 mL micro centrifuge tube maintained at 37 °C in a shaker bath. The incubation mixture contained chlorzoxazone (10–800 µM; Sigma-Aldrich), the microsomal fraction of COS-7 cells (5 mg/100 µL protein final concentration) and a NADPH-generating system (1 mM NADP^+^, 10 mM glucose 6-phosphate, 5 mM MgCl_2_, and 2 U/mL glucose 6-phosphate dehydrogenase; Promega https://www.azbiochem.com) in 0.1 M potassium phosphate buffer (pH 7.4) at a final volume of 200 µL. The reaction was initiated by adding the NADPH-generating system after preincubation at 37 °C for 5 min. Then, after 30 min incubation with chlorzoxazone 6-hydroxylation and gentle shaking, the reaction was terminated by adding 50 µL of cold acetonitrile followed by one mL of ethyl acetate. The mixtures were spiked with 1 mg/mL phenacetin (Sigma-Aldrich) as an internal standard and vigorously vortexed for 2 min. After centrifugation at 2000 g for 20 min, the organic phase was evaporated to dryness under a gentle stream of nitrogen at 35 °C. The residues were dissolved in methanol-water (50:50, v/v) and filtered with a polytetrafluoroethylene membrane filter (13 mm, 0.45 µm; Dikma Technologies, Lake Forest, CA, USA), and 20 µL of the filtrate was subjected to HPLC analysis. Every reaction was performed in triplicate.

The HPLC analysis was performed using the Thermo Scientific™ Ultimate™ 3000 system (Thermo Fisher Scientific, Shanghai, China). The cooled samples were separated on a Thermo Accucore-C18 column (4.6 mm ×150 mm, 2.6 µm) and maintained at 25 °C. The product 6-hydroxychlorzoxazone (Sigma-Aldrich) in the assay for chlorzoxazone 6-hydroxylation activity and the internal standard phenacetin were eluted isocratically with HPLC-grade 0.6% glacial acetic acid and acetonitrile (30:70, v/v) as the mobile phase at a flow rate of 1.0 mL/min. UV detection was performed at 290 nm. Under these conditions, the retention times of 6-hydroxychlorzoxazone and phenacetin was 5.0 and 2.8 min, respectively. The standard curve samples for assay was prepared in the same manner as the incubation samples. The lower limit of 6-hydroxychlorzoxazone quantification was 2.4 nM. An eight-point standard curve was used to quantify the amount of 6-hydroxychlorzoxazone in the samples.

### In silico analysis and molecular structural modeling

To determine the potentially deleterious effect of the amino acid changes, three publicly available variant effect prediction tools, i.e., Polymorphism Phenotyping v2 (Polyphen 2, http://genetics.bwh.harvard.edu/pph2/, Protein Variation Effect Analyzer (PROVEAN, http://provean.jcvi.org/index.php and Sorting Intolerant From Tolerant (SIFT, http://sift.jcvi.org), were used. Polyphen 2 is designed to predict whether an amino acid substitution will affect protein function based on high-quality multiple sequence alignment with a probabilistic classifier based on machine-learning, and the results were divided into 3 categories: benign (0.00 to 0.15), possibly damaging (0.15 to 0.85), and probably damaging (0.85 to 1.00), with scores ranging from 0 to 1 (Adzhubei et al., 2010). The effect of amino acid substitution or indels on the biological function of a protein based on the 3D protein model were predicted by PROVEAN software and the results were divided into 2 categories: deleterious (−14 to −2.5) and neutral (−2.5 to 14), with scores ranging from −14 to 14 (Choi & Chan, 2015). SIFT is a software tool that uses the sequence homology and the physical properties of amino acids to predict the effect of amino acid substitution on protein function, and the results were divided into 2 categories: damaging (0 to 0.05), and tolerated (0.05 to 1.00), with scores ranging from 0 to 1 (Sim et al., 2012). The web server versions of these tools were used under their default settings. We performed three-dimensional (3D) protein modelling of mutational and wild-type proteins, and the protein structure was visualized by Swiss-PDB Viewer (Guan et al., 2018).

### Statistics analysis

Protein expression data and enzyme activity analyses were performed using GraphPad Prism 5.0 software (GraphPad Software, San Diego, CA, USA). Pearson correlations were used to evaluate the associations between the CYP2E1 protein content and enzyme activity with two-sided *t*-test. Enzyme kinetic parameters of different CYP2E1 variants toward 6-hydroxychlorzoxazone were calculated using a non-linear regression based on the Michaelis–Menten equation. Statistical comparisons were performed using GraphPad Prism 5.0, with Dunnett t-tests for mean comparisons for each variant genotype against the wild-type control. The results were considered statistically significant at *P* < 0.05.

## Results

### Changes in the CYP2E1 protein expression of CYP2E1 variants

**Figure 1:**
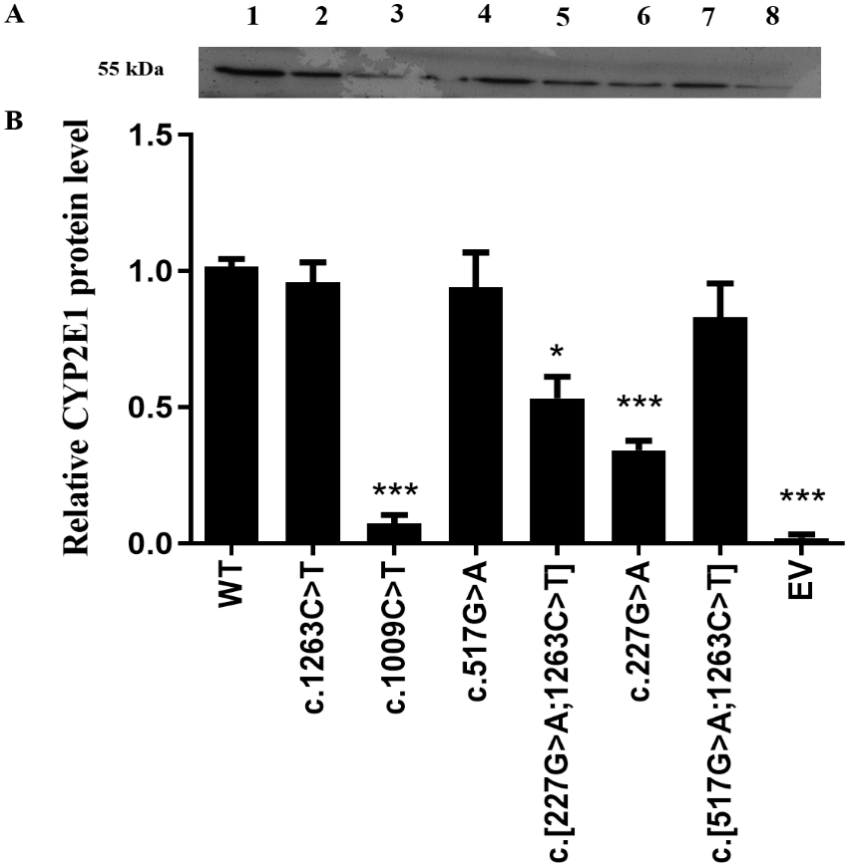
Detection of CYP2E1 proteins by western-blot analysis. Detection of CYP2E1 proteins by western-blot analysis. (A) Recombinant CYP2E1 variants as well as the wild type (WT) and empty vector (EV) as a negative control were recognized by rabbit-produced anti-CYP2E1 antibody. The loading amount was 10 µg microsomal protein. All lanes are as shown above. 1, WT; 2, c.1263C>T; 3, c.1009C>T; 4, c.517G>A; 5, c.[227G>A;1263C>T]; 6, c.227G>A; 7, c.[517G>A;1263C>T]; 8, EV. (B) Relative expression levels of CYP2E1 variants. The results are expressed as a percentage of the WT. All values are mean +/- S.E.M for three separate independent assays. ^∗^*P* ≤ 0.05,^∗∗^*P* ≤ 0.01,^∗∗∗^*P* ≤ 0.001.

Recombined expression plasmids carrying the desired mutants were identified by direct DNA sequencing (Fig. S1), and the sequencing primers for allelic variants are provided in Table S1. As a first step in our functional analysis, we investigated whether the CYP2E1 protein expression and stability were affected by the genetic variation in encoded amino acid sequence. All recombinant CYP2E1variants as well as the wild type (WT) and empty vector (EV) as a negative control were expressed in COS-7 cells, and their relative protein levels were measured by western blot analysis (Fig. 1). The differences of CYP2E1 protein between the WT and variants were considered statistically significant at *P* < 0.05. As shown in Fig. 1A, all variants were immunodetectable with the rabbit-produced anti-CYP2E1 antibody. Compared with the WT (the WT of CYP2E1), the protein expression levels of the three allelic variants c.1009C>T, c.227G>A and c.[227G>A;1263C>T] were reduced by 97.2%, 65.6%, and 43.7% (*P* < 0.05), respectively. Although the western blot analysis of protein expression showed reduced expression of the CYP2E1 protein for *CYP2E1* variant c.517G>A relative to that of the WT, the difference was not statistically significant. The CYP2E1 protein expression level of *CYP2E1* allelic variants c.1263C>T and c.[517G>A;1263C>T] were comparable to that of the WT (Fig. 1B). Overall, western blot analysis of protein expression showed reduced protein levels of CYP2E1 variant allozymes.

### Changes in enzyme activity in CYP2E1 variants

**Figure 2:**
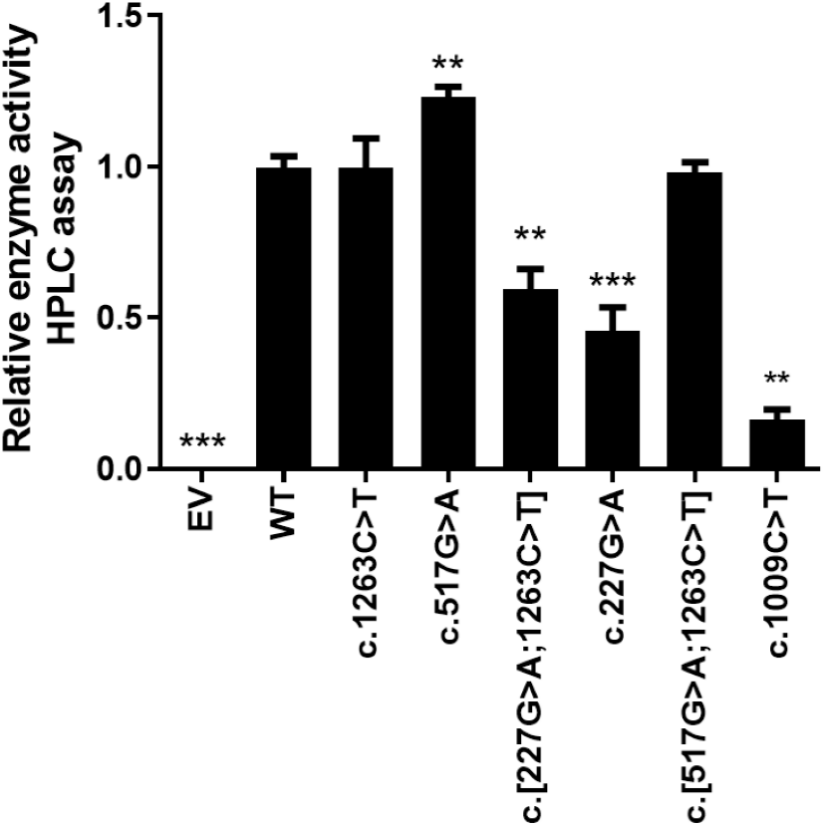
Relative CYP2E1 Enzyme Activity. Bar graphs showing normalized enzyme activity of the CYP2E1 wild type and variant enzymes (Each bar represents the mean ± SEM of three separate independent experiments). Enzyme activity was analyzed using one-way ANOVA, with mean comparison for each variant enzyme against the wild-type control performed using Dunnett’s test. ^∗^*P* ≤ 0.05,^∗∗^*P* ≤ 0.01,^∗∗∗^*P* ≤ 0.001.

**Figure 3:**
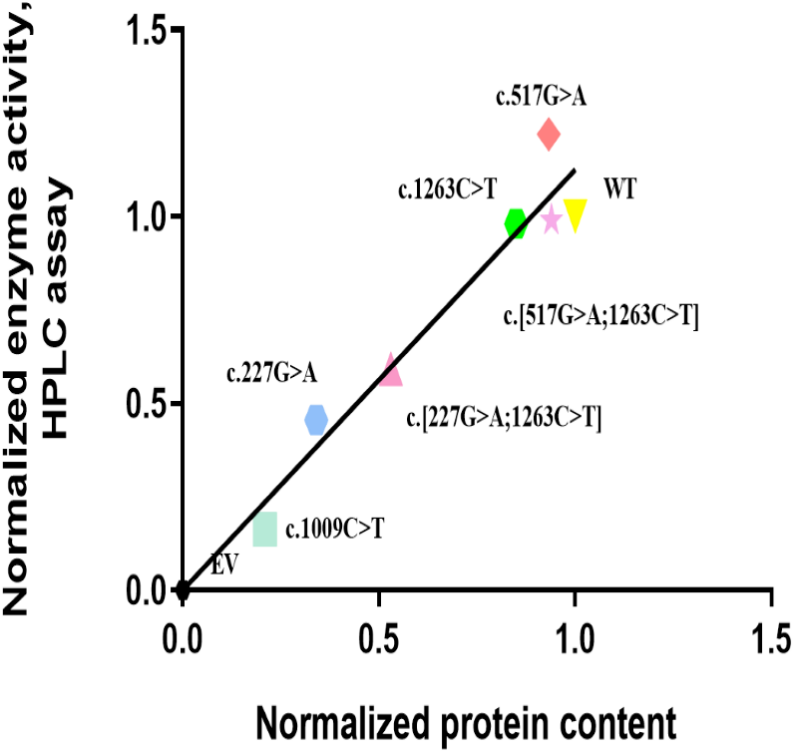
Correlation analysis of recombinant protein quantity and enzyme activity for CYP2E1 variant allozymes. The Normalized protein quantities are plotted on the horizontal axis and the Normalized enzyme activities are plotted on the vertical axis. r^2^ = 0.8833 and *p* value = 0.0005. Pearson correlations was used to evaluate the associations between the CYP2E1 protein content and enzyme activity, with two-sided *t*-test.

The *in vitro* enzyme activity levels of COS-7 cell-expressed CYP2E1 toward chlorzoxazone 6-hydroxylation were determined using chlorzoxazone as a substrate, with experiments conducted in triplicate, and the results are shown in Fig. 2. Meanwhile, the correlations between the enzyme activity and protein expression studies (Fig. 1B) for CYP2E variant enzymes were investigated. Positive correlations were observed between the protein content and enzyme activity for CYP2E1 (*p* = 0.0005; *r*^2^ = 0.8833), as shown in Fig. 3. However, there were variants displayed inconsistent trends in protein expression and enzyme activity. For example, c.517 G>A variant showed increased enzyme activity, but its protein expression level was comparable to that of the WT enzyme. These observations suggest that enzyme activity for several of the variant enzymes were significantly affected by protein expression.

**Figure 4:**
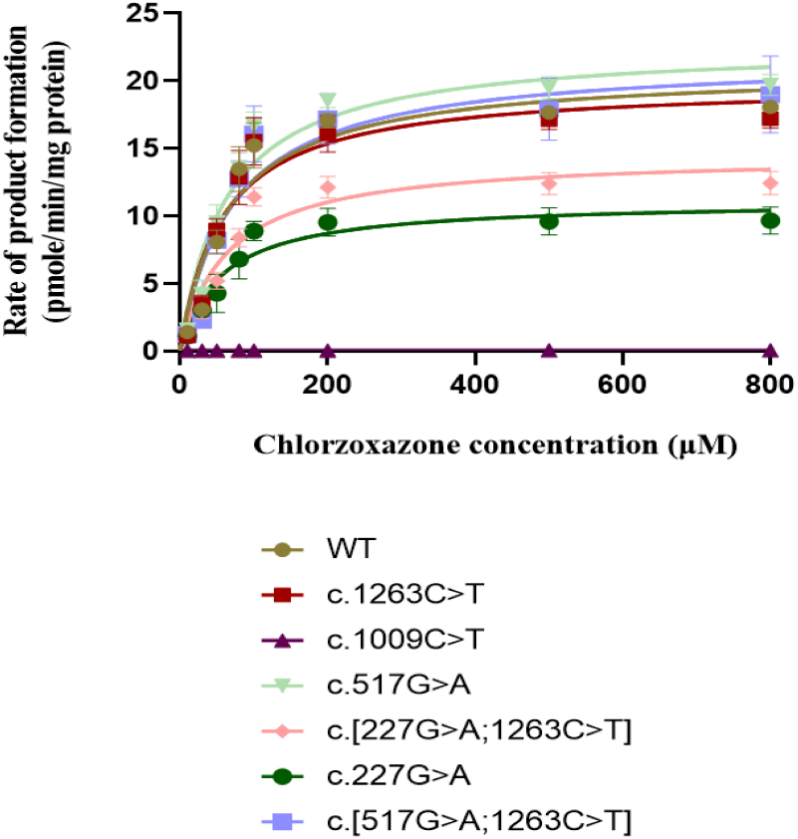
Michaelis Menten curves of chlorzoxazone 6-hydroxylation by the COS-7 cell-expressed recombinant wild-type and six variants of CYP2E1 enzymes (each point represents the mean ± SEM of three separate independent experiments). Enzyme kinetic were modeled using a non-linear regression based on the Michaelis-Menten equation.

The concentration range of product 6-hydroxychlorzoxazone formation in samples for CYP2E1 was 0–30 nM. As shown in Fig. 4, all recombinant CYP2E1s presented typical Michaelis–Menten kinetic profiles for chlorzoxazone metabolism. The enzyme metabolic kinetic parameters for recombinant CYP2E1variant enzymes as well as WT were summarized in Table 2, with data shown in the form of mean ± S.D. The kinetic parameters of *K*_*m*_, *V*_*max*_, and intrinsic clearance (*CL*_*int*_) for the COS-7 cell-expressed WT CYP2E1 (WT) enzyme toward chlorzoxazone 6-hydroxylation were 63.47 µM, 21.75 pmol/min/pmol CYP2E1 and 0.3841 µL/min/pmol CYP2E1, respectively. For *CYP2E1,* we found that the *V*_*max*_ values of variants were significantly decreased for c.227G>A and c.[227G>A;1263C>T] (reduced by 40.3% and 33.1%, respectively; *P* < 0.05) when compared to that of the WT enzyme. The *V*_*max*_ values for chlorzoxazone metabolism of c.1263C>T and c.[517G> A;1263C>T] variant enzymes were comparable to that of the WT. Relative to the WT enzyme, the recombinant c.517G>A exhibited significantly lower *K*_*m*_ value (reduced by 16.1%; *P* < 0.05). The *K*_*m*_ value of c.1263C>T allelic variant was reduced by 10.2% of the WT enzyme, that did not achieve statistical significance. For *CYP2E1,* we found that the *CL*_*int*_ value of variants were less than 70% (low enzyme activity) for c.227G>A, c.[227G>A;1263C>T], ranged from 90–100% (equal or similar to WT) for c.1263C>T, c. [517G>A;1263C>T] and more than 100% (high activity) for c.517G>A when compared to that of the WT enzyme. Overall, for *CYP2E1*, we found that the catalytic activities of variants were significant difference for c.227G>A, c.[227G>A;1263C>T], and c.517G>A when compared to that of the WT enzyme.

**Table 2:** Calculated Michaelis-Menten parameters for chlorzoxazone 6-hydroxylation by wild-type and variant forms of CYP2E1. Each value is the mean ± SD of three independent experiments. Vmax represents the maximal rate at which the enzyme metabolizes the substrate; Km represents the Michaelis constant; and the Clint value represents the intrinsic clearance. Three online software programs were simultaneously used to predict genetic mutations. The three algorithms predicted no effect (benign/tolerant/neutral) or loss of function (damaging/probably damaging/deleterious). When the results of two of the three software predictions were consistent with the results of the HPLC experiment, the results were considered to be consistent, and we added YES; otherwise, we added NO.

**Variant**	***V***_***max***_**± SD** **(pmol/min/pmol CYP2E1)**	***K***_***m***_**± SD** **(µM)**	***CL***_***int***_ **(µL/min/pmol CYP2E1)** **(% of wild-type)**	**Polyphen**	**SIFT**	**PROVEAN**	**Concordance** **Y/N**
Wild-type	21.75 ± 1.413	63.47 ± 13.81	0.3841 ± 0.0631				
c.1263C>T	21.65 ± 1.393	56.95 ± 12.67	0.4027 ± 0.052 (102%)	N/A	Tolerated	Neutral	YES
c.1009C>T	0.02			N/A	N/A	Deleterious	N/A
c.[517G>A+1263C>T]	21.34 ± 1.29	60.81 ± 12.46	0.379 ± 0.078 (98.6%)	N/A	N/A	N/A	N/A
c.517G>A	23.06 ± 1.354	53.2 ± 11.03^*^	0.511 ± 0.089 (131%)^**^	probably damaging	Tolerated	Deleterious	YES
c.[227G>A+1263C>T]	14.54 ± 0.8015^**^	64.09 ± 11.82	0.258 ± 0.055 (67.2%)^**^	N/A	N/A	N/A	N/A
c.227G>A	12.96 ± 0.74^**^	65.8 ± 12.45	0.22 ± 0.041 (57.2%)^**^	probably damaging	Damaging	Neutral	YES

**Notes.**

* *P* ≤ 0.05.

** *P* ≤ 0.01.

*** *P* ≤ 0.001.

### In silico genetic variation prediction analysis

The effect of *CYP2E1* genetic variation in coding region on protein function was predicted using three programs: Polyphen 2, PROVEAN and SIFT (Table 2). For *CYP2E1*, we found that the protein functions of variants were predicted to have no effect (N/A/neutral/tolerated) for the c.1263C>T variant, a loss of function (probably damaging, deleterious, tolerated) for the c.517G>A variant, and a loss of function (probably damaging, damaging, neutral) for the c.227G>A variant using the three programs. Concordance was defined as consistency between least two of three prediction tool results and the enzyme catalytic assay result. The results suggest that there was high consistency between programs and functional assay results under the condition that all prediction software performed functional assessments of the variants that altered encoded amino acid sequences. Furthermore, we found that the protein function of the c.1009C>T variant was not predicted with Polyphen 2 and SIFT because these two programs are not suitable for the prediction of nonsense mutations (translational stop codon). We also found that *in silico* tools were not applicable to predict the functional impact of the combination allelic variants c.[227G>A;1263C>T] and c.[517G>A;1263C>T]. Overall, these results suggest that the *CYP2E1* missense variants (c.227G>A and c.517G>A) in the coding region would affect protein function.

### Molecular structural modeling

**Figure 5:**
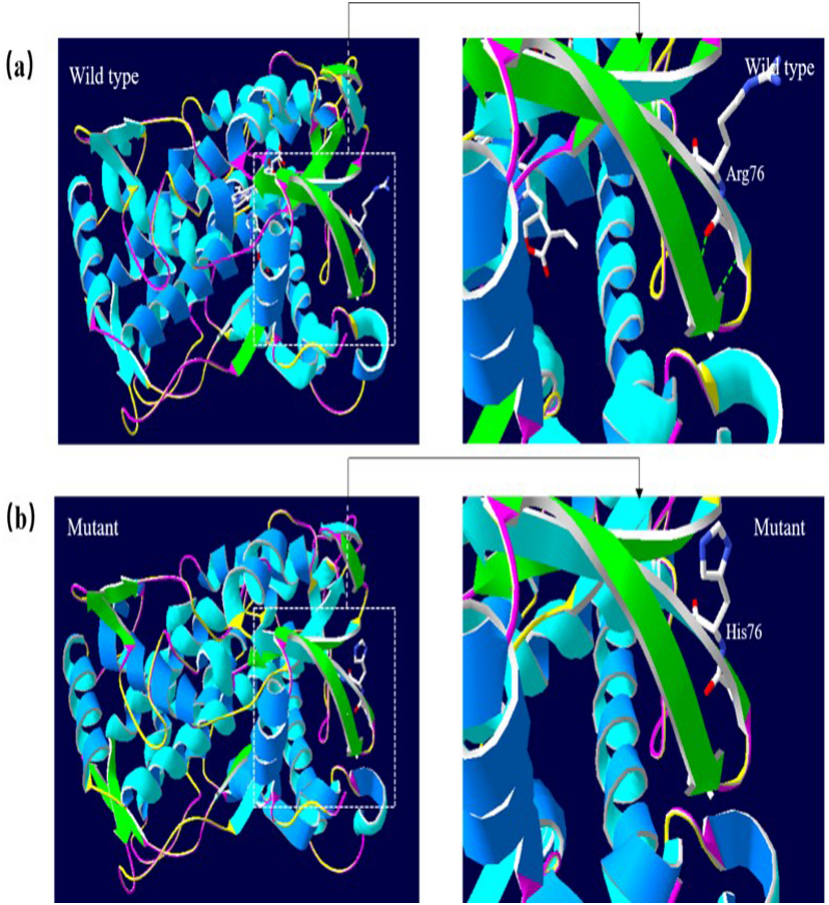
3D homologous molecular modeling comparison of the wild type and mutant (p.R76H, Arg76 to His) in the CYP2E1 protein. (A) Wild-type protein has a H-bond between Val72 and Gln75. (B) Mutant protein His76 is predicted to lose the H-bond between Val72 and Gln75 due to the substitution of arginine to histidine at position 76, which may perturb the amino acid side chain. Comparison sites are highlighted by white frame lines and locally zoomed.

**Figure 6:**
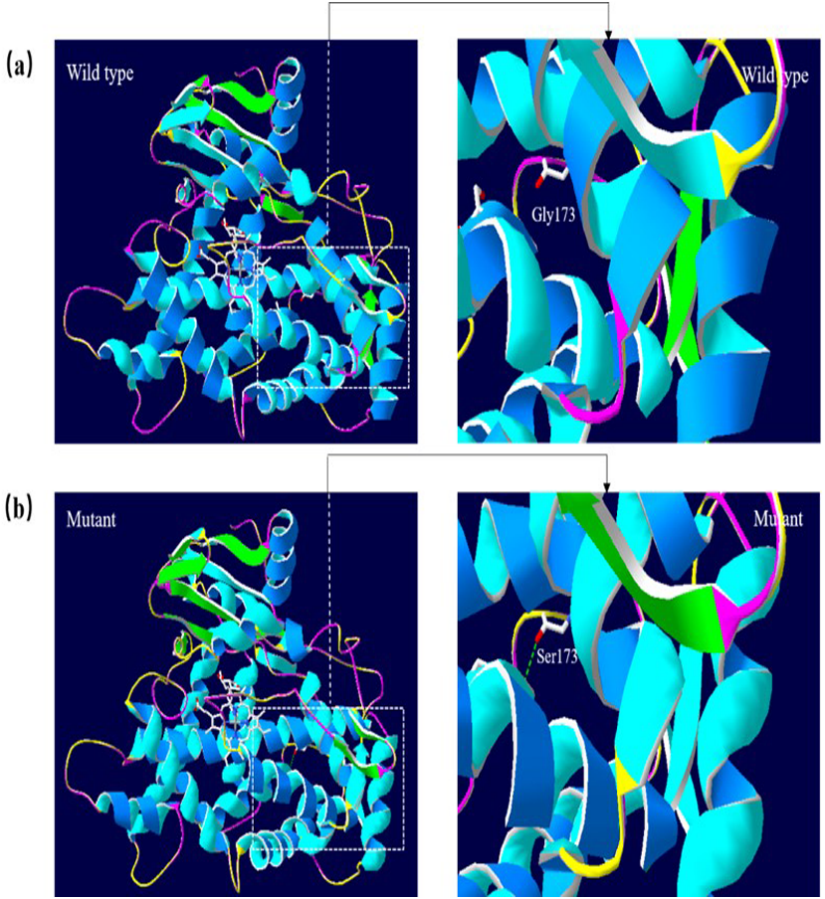
Molecular modeling comparison of the wild type and missense mutant (p.G173S, Gly173 to Ser) in the CYP2E1 protein. (A) Wild-type protein has no H-bond between the Pro168 and Ile172. (B) Mutant protein Ser173 is predicted to increase the H-bond between Pro168 and Ile172 due to the substitution of glycine to serine at position 173, which possibly changed the orientation of the amino acid residues. Comparison sites are highlighted by white frame lines and locally zoomed.

A 3D structural model of CYP2E1 was generated with SWISS-MODEL to determine whether the p.Arg76His (c.227G>A) and p.Gly173Ser (c.517G>A) missense mutations affect the structures of CYP2E1. The p.Arg76His mutation was predicted to result in the loss of a hydrogen bond between Val72 and Gln75 due to the substitution of arginine to histidine at position 76, possibly perturbing the amino acid side chain and affecting the structure of CYP2E1(as visualized in Fig. 5). Using Swiss-Pdb Viewer 4.1, the p.Gly173Ser mutation was predicted to result in the increase of a hydrogen bond between the Pro168 and Ile172, which possibly changed the orientation of the amino acid residues and affected the structure of the protein (Fig. 6). These observations suggest that the amino acid changes would affect the structures of CYP2E1 for variants c.227G>A and c.517G>A.

## Discussion

The drug-metabolizing enzyme encoded by the *CYP2E1* gene is a liver microsomal enzyme that plays an important role in drug metabolism and toxicity susceptibility assessment (Kim & O’shea, 1995). Large interindividual variation in the activity both *in vivo* and *in vitro* has been reported, and genetic polymorphisms in CYP2E1 have been considered important contributors to this interindividual diversity (Lucas et al., 1995), (Song, 1996). Since the polymorphisms in the CYP2E1 gene would influence protein expression and the enzyme activity of allozymes encoded by the variant sequences, it has been suggested to be an important risk factor for susceptibility to several cancers. Therefore, the assessment of the metabolic capacity of *CYP2E1* variants is particularly important to the prediction of the metabolic activity of drugs *in vivo* and xenobiotic inactivation (Song, 1996; Tanaka, Terada & Misawa, 2000). In our study, we focused on six alleles (c.1009C>T, c.227G>A, c.517G>A, c.1263C>T, c.[227G>A;1263C>T], and c.[517G>A;1263C>T]) that were identified in the Han Chinese population. We expressed recombinant CYP2E1 enzymes of these variants and the WT in COS-7 cells and investigated their enzyme activity via a kinetic analysis using chlorzoxazone as a probe. The expression of CYP2E1 variants after transfection into COS-7 cells most often yielded differing protein levels for the variants’ enzymes as compared to the WT sequence. Using chlorzoxazone as a probe to assess the impact of these variants on biological function, we observed that there were also significant differences in enzyme activity among the variants that were normally associated with protein expression levels. We also found that there was a positive correlation between the protein expression level and enzyme activity in each variant of CYP2E1. These observations suggest that reduced protein expression levels may be the main cause of reduced enzyme activity, which is consistent with previous findings that protein degradation resulted from misfolding due to amino acid sequence substitution (Wang et al., 2005; Li et al., 2008).

In this study, an immunoblot analysis was used to quantitatively detect the expression of all recombinant CYP2E1enzymes, which is a method that has been widely used in the *in vitro* detection of the expression levels of CYP superfamily proteins (Marcucci et al., 2002; Watanabe et al., 2010, Hiratsuka, 2012; Du et al., 2016). In brief, the microsomal fractions containing the expressed proteins were subjected to immunoblot analysis, with three independent biological repeats. We found that the expression levels of the CYP2E1 protein from c.1009C>T and c.227G>A variants resulted in p.Arg337X and p.Arg76His amino acid substitutions, respectively, and were significantly lower than that of the WT (<50%). Among these variants, c.227G>A, which yields p.Arg76His and is termed CYP2E1*2, has been reported most extensively. Hu et al. (1997) reported that the protein level of this variant expressed in COS-1 cells was decreased to 37% of that of the WT, although the amounts of mRNA were very similar (Hu et al., 1997). Hanioka et al. (2003) also found that the protein content of this allelic variant was reduced to 29% compared with that of the WT, while the transcription and translation levels were not affected (Hanioka et al., 2003). In addition, Hanioka et al. (2010) reported that CYP2E1*2 cDNA reduces the expression level of protein (37% of CYP2E1*1 cDNA) using yeast cells (Hanioka et al., 2010). Thus, the expression profile of CYP2E1 obtained in an expression system using COS-7 cells was consistent with those of previous studies and the decreased protein level of CYP2E1*2 may be closely related to the change of protein stability or folding efficiency.

Chlorzoxazone is widely used as a probe substrate to evaluate the catalytic capacity of the CYP2E1 enzyme by determining the kinetic parameters of chlorzoxazone 6-hydroxylation *in vivo* and *in vitro* (Lucas, Menez & Berthou, 1996; Howard et al., 2001). The enzyme activity assay performed in this study used pmol/min/pmol CYP2E1 to define the enzyme activity of each variant and WT CYP2E1 enzyme (Du et al., 2016). The analysis of enzyme activity showed that two of the six CYP2E1 variant enzymes exhibited an equal catalytic capacity to the WT (90–100%), one variant enzyme showed a higher metabolic capacity (100–200%), and the other three variant enzymes exhibited lower enzyme activity (<50% of WT). Notably, the enzyme activity based on the microsomal protein concentration of the c.227G>A variant, which resulted in an p.Arg76His amino acid substitution, was decreased relative to that of the WT. Hu et al. (1997) reported that CYP2E1*2 cDNA only caused 36% of the catalytic activity toward chlorzoxazone 6-hydroxylation compared with the WT cDNA in an expression system using COS-1 cells (Hu et al., 1997). Hanioka et al. (2003) also reported that the oxidation activities of CYP2E1*2 for chlorzoxazone 6-hydroxylation and 4-nitrophenol 2-hydroxylation were significantly lower than those of WT CYP2E1 (Hanioka et al., 2003). Interestingly, we also observed a significant correlation between protein expression levels and enzyme activities for recombinant CYP2E1 enzymes. Our results suggest that the decreased protein level as a result of *CYP2E1* polymorphisms may be an important contributor to the decreased enzyme activity for the variant enzyme.

The microsomal fractions in COS-7 cells containing the expressed proteins were further used to determine the enzymatic kinetic parameters *K*_*m*_*, V*_*max*_*,* and *CL*_*int*_ (µL/min/pmol CYP2E1) to evaluate the function of all variants and WT CYP2E1 enzymes. We confirmed that the kinetics of chlorzoxazone 6-hydroxylation in microsomal fractions containing the expressed proteins fit a single-enzyme model with a typical Michaelis–Menten equation, and the obtained *K*_*m*_ was 55.35 µM for the WT CYP2E1. Previous studies have reported microsomal oxidation values of chlorzoxazone 6-hydroxylation associated with the WT CYP2E1 expressed in human B-lymphoblastoid cells, COS-1 cells and insect cells of 81, 283, and 660 µM, respectively (Fairbrother et al., 1998; Shimada, Tsumura & Yamazak, 1999; Hanioka et al., 2003). These results were different from ours, and a possible reason for this disparity is the differences in heterologous proteins expressed in different cell lines. The *V*_*max*_ values of the c.1009C>T, c.227G>A, and c.[227G>A;1263C>T] variant enzymes with p.Arg337X and p.Arg76His amino acid substitutions, were significantly reduced compared with the WT CYP2E1 expressed in COS-7 cells. Similarly, these variant enzymes with chlorzoxazone as the representative substrate exhibited a lower *CL*_*int*_ value relative to that of WT CYP2E1. In contrast, the *CYP2E1* variant c.517G>A resulted in a p.Gly173Ser substitution and presented a 30% increase in *CL*_*int*_ compared with the WT CYP2E1. Zhu et al. (2018) also predicted the functional impact of this variant and found that the genetic variation in the structure and function of the protein may be harmful (Zhu et al., 2018). Kim & O’shea (1995) reported that there was a 10-fold increase in the amount of transcription of this variant *in vitro*, although the protein expression and enzyme activity has not been explored (Kim & O’shea1, 1995). In addition, *CYP2E1* variant c.1009C>T was predicted by PROVEAN software to be deleterious as a result of genetic variation, which is consistent with functional studies showing that a chlorzoxazone substrate was inactive toward chlorzoxazone 6-hydroxylation (Choi & Chan, 2015). Thus, these findings suggest that the p.Arg337X, p.Gly173Ser and p.Arg76His substitutions are of great importance for the catalytic activity of the CYP2E1 protein.

A large number of programs, including Polyphen 2, SIFT and PROVEAN, have been designed to predict the effect of genetic variation on protein function; however, our results support the importance of functional studies as the “gold standard” for the functional assessment of allelic variants that alter encoded amino acid sequence (Min et al., 2016). SWISS-MODEL has been widely applied to perform detailed alignment and generate homology models for the localization of amino acids in the three-dimensional structure of the enzyme (Biasini et al., 2014; Faure et al., 2019). Moreover, the biological functions of proteins often depend on their amino acid sequences and chemical bonds and the importance of hydrogen bonds during the evolution of protein interfaces has been highlighted (Nishio et al., 2014), (Kuroda & Gray, 2016). Previous studies have predicted the location of Arg76 of human CYP2E1 using the crystal structures of bacterial P450s as templates and found that this amino acid residue is conserved in all mammals and possibly has an important function (Hu et al., 1997). Furthermore, the *CYP2E1* gene has been reported to play a role in the toxicity of therapeutic drugs and environmental toxicants, which are generally considered to result from lipid peroxidation and free radical production (Song, 1996). Therefore, the results of in silico prediction and protein structure homology modeling would provide important information for protein functional impacts caused by amino acid substitution.

The standard method to determine the effect of genetic variation on CYP enzyme function involves transfection of cDNA of alleles into COS cells to express the corresponding variant enzyme and then functional characterization of their catalytic activity by kinetic analysis (Wang et al., 2005; Watanabe et al., 2010; Du et al., 2016; Devarajan et al., 2019). The CYP2E1 variant enzymes expressed in this expression system are closer to the biological activity of the enzyme itself. The results of the enzyme kinetics measured by the probe substrate are more clinically relevant than that of other expression systems. However, there are still limitations of this study: in the functional characterization experiment conducted *in vitro*, only chlorzoxazone as a representative substrate was used to determine the enzyme kinetics parameters to characterize the enzyme activity of variant enzymes. Therefore, in the future, additional substrates of CYP2E1 enzyme-mediated metabolism should be included to strengthen our results.

## Conclusions

In summary, we focused on six *CYP2E1* alleles (c.227G>A, c.1009C>T, c.517G>A, c.1263C>T, c.[227G>A;1263C>T], and c.[517G>A;1263C>T]) identified in the Han Chinese population, expressed all of them as well as the WT enzyme in COS-7 cells, and then characterized enzyme kinetics, with chlorzoxazone as the representative substrate. An individual carrying the *CYP2E1* variant c.227G>A and c.[227G>A;1263C>T] that causes p.Arg76His amino acid substitution showed lower enzyme activity in CYP2E1-mediated substrate metabolism, while an individual carrying c.517G>A that yields p.Gly173Ser change showed higher enzyme activity. The prematurely terminated p.Arg337X variant enzyme was undetectable by western blotting and inactive toward chlorzoxazone 6-hydroxylation. Therefore, we must consider the effects of the p.Arg76His, p.Gly173Ser, and p.Arg337X amino acid substitutions on the catalytic activity of CYP2E1 variant enzymes. The information gained in this study should help to promote a better understanding of CYP2E1-mediated drug metabolism.

##  Supplemental Information

10.7717/peerj.9628/supp-1 Supplemental Information 1 DNA sequencing comparison of the wild type and SNP s of the *CYP2E1* gene were identified in the Han Chinese population DNA sequence chromatogram alignment analysis shows that clones carrying the desired mutants were successfully identified by direct DNA sequencing: (A) missense mutation (c. 227G \begindispformula > \enddispformula A), (B) nonsense mutation (c.1009C \begindispformula > \enddispformula T), (C) missense mutation (c.517G \begindispformula > \enddispformula A), and (D) silent mutation (c.1263C \begindispformula > \enddispformula T). Comparison sites for each SNP variant are highlighted by red frame lines.

10.7717/peerj.9628/supp-2 Supplemental Information 2 Primers used for site-directed mutagenesis All primers were 5’-phosphorylated.

10.7717/peerj.9628/supp-3 Supplemental Information 3 Primers used for site-directed sequencing validation All primers were 5’-phosphorylated.

## Additional Information and Declarations

### Data Availability

The following information was supplied regarding data availability: The raw measurements are available in a Supplementary File.
