# Supplementary figures and images for: Functional characterization of the chlorzoxazone 6-hydroxylation activity of human cytochrome P450 2E1 allelic variants in Han Chinese
Source: PeerJ. 2020 Jul 31;8:e9628. doi: 10.7717/peerj.9628 (PMC7397980; doi:10.7717/peerj.9628)

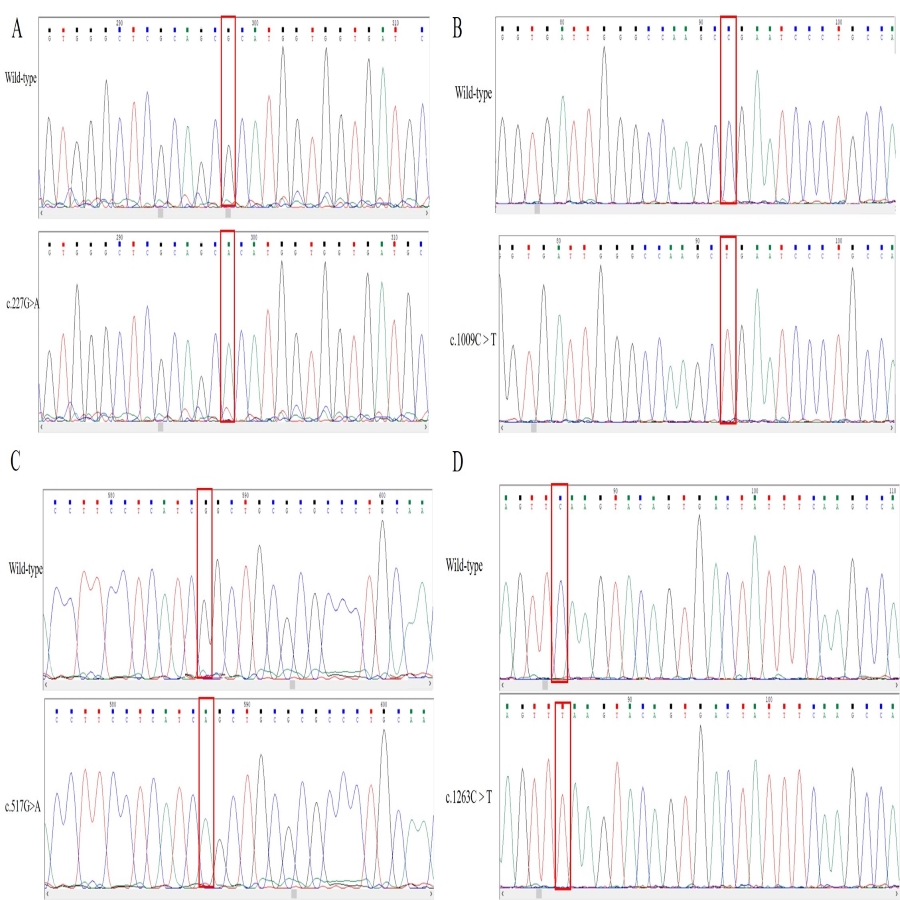

Supplement: Supplemental Information 1 — DNA sequence chromatogram alignment analysis shows that clones carrying the desired mutants were successfully identified by direct DNA sequencing: (A) missense mutation (c. 227G \begindispformula > \enddispformula A), (B) nonsense mutation (c.1009C \begindispformula > \enddispformula T), (C) missense mutation (c.517G \begindispformula > \enddispformula A), and (D) silent mutation (c.1263C \begindispformula > \enddispformula T). Comparison sites for each SNP variant are highlighted by red frame lines. [file peerj-08-9628-s001.jpg]
