# Supplementary material for: Functional characterization of the chlorzoxazone 6-hydroxylation activity of human cytochrome P450 2E1 allelic variants in Han Chinese
Source: PeerJ. 2020 Jul 31;8:e9628. doi: 10.7717/peerj.9628 (PMC7397980; doi:10.7717/peerj.9628)
Supplement: Supplemental Information 2 — All primers were 5’-phosphorylated. [file peerj-08-9628-s002.docx]

**Supplementary Table 1**

**Primers used for site-directed mutagenesis.**

| Location | Primmer | Sequence |
| --- | --- | --- |
| G227A | F | 5'GTACGTGGGCTCGCAGCATATGGTGGTGATGCACG3' |
|  | R | 5'CGTGCATCACCACCATATGCTGCGAGCCCACGTAC3' |
| G517A | F | 5' CCTTCCTCATCAGCTGCGCGCCC 3' |
|  | R | 5' GGGCGCGCAGCTGATGAGGAAGG 3' |
| C1009T | F | 5'GGTGATTGGGCCAAGCTGAATCCCTGCC3' |
|  | R | 5'GGCAGGGATTCAGCTTGGCCCAATCACC3' |
| C1263T | F | 5' GAAAATGGAAAGTTTAGTACAGTGAC 3' |
|  | R | 5' GTCACTGTACTAAACTTTCCATTTTC 3' |

All primers were 5’-phosphorylated.
