# Supplementary material for: Functional characterization of the chlorzoxazone 6-hydroxylation activity of human cytochrome P450 2E1 allelic variants in Han Chinese
Source: PeerJ. 2020 Jul 31;8:e9628. doi: 10.7717/peerj.9628 (PMC7397980; doi:10.7717/peerj.9628)
Supplement: Supplemental Information 3 — All primers were 5’-phosphorylated. [file peerj-08-9628-s003.docx]

**Supplementary Table 2**

**Primers used for site-directed sequencing validation**

| cDNA | Primers |
| --- | --- |
| 227G>A | F:5’ CCTTTCCCGCTTCCCATCAT 3’  R:5’ ATAGTTCCGGAGGGTGGTCA 3’ |
| 517G>A | F:5’ CTCCGGAACTATGGGATGGG 3’  R:5’ CCAGGGAGTGCTGAGTAGGT 3’ |
| 1009C>T | F:5’ TTCTTTGCGGGGACAGAGAC3’  R:5’ TCATGCACCACAGCATCCAT3’ |
| 1263C>T | F:5’CCCAAGGGCACAGTCGTAG3’  R:5’CTTCTCCAGCACACACTCGT3’ |
| 227G>A+1263C>T | F1:5’ CCTTTCCCGCTTCCCATCAT 3’  R1:5’ ATAGTTCCGGAGGGTGGTCA 3’  F2:5’CCCAAGGGCACAGTCGTAG3’  R2:5’CTTCTCCAGCACACACTCGT3’ |
| 517G>A+1263C>T | F1:5’ CTCCGGAACTATGGGATGGG 3’  R1:5’ CCAGGGAGTGCTGAGTAGGT 3’  F2:5’CCCAAGGGCACAGTCGTAG3’  R2:5’CTTCTCCAGCACACACTCGT3’ |

All primers were 5’-phosphorylated.
